# Enhancement of Nutritional Value and Sensory Characteristics of Quinoa Fermented Milk via Fermentation with Specific Lactic Acid Bacteria

**DOI:** 10.3390/foods14081406

**Published:** 2025-04-18

**Authors:** Li Zhao, Juan Liao, Tingyu Wang, Haijiao Zhao

**Affiliations:** College of Food Science and Engineering, Shanxi Agricultural University, Jinzhong 030801, China

**Keywords:** quinoa, LAB fermentation, PCA analysis, quinoa fermented milk, flavor substance, storage stability

## Abstract

Quinoa has garnered significant attention for its richness in a variety of nutritional and functional components. Herein, quinoa was fermented with individual or a combination of various lactic acid bacteria (LAB) strains to assess the impact of fermentation on nutrients, functional components, and digestibility. The results indicate that specific LAB fermentation significantly decreased the starch and dietary fiber content while markedly increasing the content and antioxidant capacity of free phenolics. The highest content of free phenolics in fermented quinoa reached 5.64 mg GAE/g, with a 2.01-fold increase in bioavailability. A comprehensive PCA evaluation identified the *MS2* mixed strain (a 1:1:1 mixture of *L. casei89*, *L. fermentum61*, and *L. rhamnosus05*) as a superior quinoa fermentation agent. Quinoa fermented milk prepared with *MS2* exhibited favorable taste and aroma properties. After 21 days of storage, the viable bacteria count remained above 10 log CFU/mL, and both the water-holding capacity and suspension stability were still strong. This study provides practical evidence for developing quinoa into a functional food.

## 1. Introduction

Quinoa (*Chenopodium quinoa* Willd.) is a highly nutritious crop rich in high-quality proteins; vitamins; unsaturated fatty acids; minerals; and various functional compounds, such as phenolics [1]. Quinoa is widely recognized as one of the superior plant-based protein sources, as its nutritional value can well meet the regulations set by the Food and Agriculture Organization (FAO). Compared with other cereals, quinoa is characterized by a harmoniously balanced amino acid composition, which is particularly rich in lysine [2]. Due to its superior protein quality, the National Aeronautics and Space Administration (NASA) has designated quinoa as an optimal food source to fulfill the nutritional requirements of astronauts during long-duration space missions [3]. Moreover, quinoa exhibits a distinct flavor profile that can enhance its sensory appeal and acceptance when incorporated into foods such as biscuits and cakes. Over 20 types of phenolic compounds have been identified in quinoa, such as caffeic acid and quercetin, which are known for their roles in regulating glucose and lipid metabolism and antioxidant properties [4]. However, these compounds in quinoa are primarily covalently bound to cellulose, hemicellulose, lignin, pectin, and proteins through ester and ether linkages and need to be released for absorption and utilization by the human body [5]. Additionally, quinoa contains high levels of insoluble dietary fiber and saponin, which have obvious negative impacts on its palatability. Various processing methods, such as milling, cooking, ultrafine grinding, roasting, and extrusion processing, have been widely used in the food industry to improve the nutritional, textural, and sensory qualities of quinoa [6]. However, the phenols in quinoa are predominantly located in the outer layers, and some processing methods may significantly damage these layers, thereby decreasing levels of phenolic compounds and antioxidant capacity. For instance, when quinoa is milled to a polish degree of 30%, the level of free and bound phenolics will decrease by 21.5% and 35.2%, respectively [7], and toasting was found to significantly decrease the overall phenolic substance levels in quinoa [8]. These negative effects may compromise the outstanding antioxidant activity and other biological properties of phenols in quinoa, such as their anti-obesity, anti-diabetic, and anti-inflammatory effects. In addition, despite its high nutritional value and wide cultivation, quinoa remains in the primary processing stage and is often marketed as a raw agricultural product with low added value and market competitiveness. Therefore, there is an urgent need to employ other processing methods for quinoa to fully utilize its potential, thereby promoting the product value and enhancing the economic, social, and ecological benefits of the industry.

Microbial fermentation, particularly probiotic fermentation, is one of the important bioprocessing techniques aimed at enhancing the safety and sensory quality of cereals and pseudocereals, such as quinoa. Probiotic fermentation can not only reduce the contents of saponins, alkaloids, oxalates, phytic acids, and tannins but also improves the digestibility of proteins and the utilization of minerals and bioactive compounds [1]. Lactic acid bacteria (LAB) strains possess the capability of fermenting glycerol and xylose and also metabolizing phenolic compounds, making them promising candidates as starter cultures in the fermentation of quinoa-based products [9]. A previous study found that fermentation of quinoa using *Lactobacillus plantarum* effectively reduces the levels of antinutritional factors, including saponins, alkaloids, oxalates, phytic acid, and tannins [10]. Another study showed that fermentation using *Lactobacillus casei* increased the thiamine, riboflavin, and free phenolic content of quinoa and decreased the content of bound phenolics after 24 h [11]. The total phenolic content (TPC) of unfermented quinoa is only about 0.11–0.18 mg GAE/100 g (DW), and solid fermentation with two strains of *Lactobacillus plantarum* significantly increased the TPC of quinoa after 72 h [12]. Therefore, fermentation using LAB strains represents a sustainable and eco-friendly technological approach to improve the comprehensive quality of quinoa. However, different LAB strains have different fermentation capabilities for quinoa, as well as different resistance capabilities to external environments and impacts on the nutrition and bioactive components of quinoa [13]. Currently, there have been few reports on the comprehensive quality evaluation system for quinoa fermented by different LAB strains and no systematic comparisons regarding the quality of quinoa fermented by single and mixed probiotics.

Therefore, this study applied six different LAB strains for both single-strain and multi-strain fermentation of quinoa and analyzed the changes in various indicators before and after fermentation, including the physicochemical properties, nutritional components, functional active components, and antioxidant activity of quinoa. Principal component analysis (PCA) was then applied to conduct a comprehensive evaluation of fermentation efficacy for screening high-quality LAB strains for quinoa. Fermented milk represents one of the most prevalently consumed fermented dairy products globally, and the fermentation process during fermented milk production generates some novel metabolites relative to milk, which confer fermented milk with unique product attributes. Hence, quinoa fermented milk was prepared using the selected fermenting strains, and the physicochemical and flavor characteristics of the quinoa fermented milk were evaluated. The results are expected to lay a solid foundation for developing quinoa into a functional food with high nutritional value and bioactivity.

## 2. Materials and Methods

### 2.1. Materials and Reagents

Quinoa were purchased from Fan Shi County Yi Kang Local Products Co., Ltd. (Xinzhou, China). *Lactobacillus bulgaricus* (*LB*), *Lactobacillus casei* (*LC*), *Lactobacillus fermentum* (*LF*), *Lactobacillus rhamnosus* (*LR*), *Lactobacillus plantarum* (*LP*), and *Lactobacillus acidophilus* (*LA*) were obtained from Jiangsu Weikang Biotechnology Co., Ltd. (Suzhou, China). The Folin–Ciocalteu reagent, sodium carbonate, gallic acid (≥98%), MRS medium, Coomassie Brilliant Blue G250, and sodium hydroxide were obtained from Shanghai Macklin Biochemical Technology Co., Ltd. (Shanghai, China). Hexane, methanol, ethanol, phenol, potassium iodide, and phosphoric acid were obtained from Tianjin Zhiyuan Chemical Reagent Co., Ltd. (Tianjin, China). 2,2-diphenyl-1-picrylhydrazyl (DPPH) and 2,2′-casino-bis (3-ethyl-benzothiazoline-6-sulphonic acid) (ABTS) were obtained from Shanghai Yuanye Biotechnology Co., Ltd. (Shanghai, China). 3,5-dinitrosalicylic acid, branched starch, and amylose were provided by Beijing Solarbio Technology Co., Ltd. (Beijing, China).

### 2.2. Strain Activation and Culture

Six kinds of LAB powder were suspended in physiological saline and individually inoculated into an MRS liquid medium for incubation at 37 °C for 24 h, with two sequential subcultures. After centrifugation at 5000 rpm for 10 min, the activated strains were washed with normal saline twice and suspended in 50 mL of sterile water. The bacteria count of each microbial suspension was standardized to 1.0 × 10^8^ CFU/mL for future use.

### 2.3. Quinoa Fermentation

Whole quinoa seeds were selected, rinsed thrice, and immersed in clean water for 24 h. The seeds were then drained for 24 h, then subjected to autoclave sterilization at 121 °C for 20 min, and subsequently cooled to room temperature for later use. The activated bacterial suspensions were inoculated into sterilized quinoa (as shown in Table 1) and fermented at 37 °C for 72 h. After fermentation, the samples were filtered using a 60-mesh sieve to obtain fermented quinoa powder for further use.

### 2.4. Color Measurement

The lightness (L*), green–red (a*), and blue–yellow (b*) color parameters of the samples were determined using a CM-5 spectrophotometer (Konica Minolta 283 Investment, Ltd., Shanghai, China). Additionally, color variation (Δ*E*) was calculated using the Equations (1)–(4) [14].

(1) $$∆E=\sqrt{{{\left({∆L}^{*}\right)}^{2}+\left(∆a^{*}\right)}^{2}+{\left({∆b}^{*}\right)}^{2}}$$

(2) $${∆L}^{*}=L_{treatment\;group}^{*}-L_{control\;group}^{*}$$

(3) $${\;\;\;\;\;\;\;\;\;∆a}^{*}=a_{treatment\;group}^{*}-a_{control\;group}^{*}$$

(4) $$\;{∆b}^{*}=b_{treatment\;group}^{*}-b_{control\;group}^{*}$$

### 2.5. Extraction and Measurement of Protein

First, 1.0 g of the quinoa sample was accurately weighed into a 50 mL centrifuge tube, to which 10 mL of n-hexane was added and mixed thoroughly. Then, the mixture was left to stand for 1 h for phase separation. The supernatant was discarded and replaced with 10 mL of distilled water. The samples were agitated at ambient temperature for 1 h. Subsequently, they were centrifuged at 5000 rpm for 10 min to separate the supernatant. After that, the albumin content in the supernatant was determined. The solid residue was re-suspended in 10 mL of a 3% NaCl solution and agitated for 1 h at room temperature before centrifugation at 5000 rpm for 10 min. The supernatant was collected for globulin quantification. Protein fractions were quantified using the Coomassie Brilliant Blue G-250 staining method and are expressed as mg BSA/g DW equivalents.

### 2.6. Extraction and Measurement of Amylose and Amylopectin

First, 100 mg of quinoa was accurately weighed into a 50 mL conical flask. Then, 1 mL of anhydrous ethanol and 9 mL of a 1 M KOH solution were introduced into the flask. The flask was sealed and immersed in boiling water for 40 min to hydrolyze the starch. After cooling, the content was diluted to a final volume of 50 mL with distilled water to form a dispersion. Then, 6 mL of this dispersion was moved into a centrifuge tube, followed via the addition of 2 mL of petroleum ether and vortexing for 5 min to facilitate lipid extraction. The phases were allowed to separate, and the petroleum ether layer was removed. The defatting process was repeated three times to ensure complete lipid removal. Then, 2 mL of the defatted solution was moved into a 50 mL volumetric glass flask, followed by the addition of 6 mL of 0.09 M KOH, 2 mL of 1 M acetic acid, and 1 mL of an iodine reagent. The solution was diluted to a final volume of 50 mL with distilled water and allowed to stand at room temperature for 30 min, and then, absorbance was determined at the wavelengths of 535 nm, 630 nm, and 757 nm. The amylose and amylopectin contents were determined using the corresponding standard curves.

### 2.7. Extraction and Measurement of Total Sugar

Initially, 1.0 g of the sample was accurately weighed and suspended in distilled water. The sample was then added with 10 mL of 6 M HCl and hydrolyzed at a boiling temperature for 30 min. The hydrolyzed solution had its pH adjusted to 7 by the addition of NaOH. Subsequently, it was diluted with distilled water to a final volume of 100 mL and then filtered. Subsequently, 1 mL of the filtrate was transferred and diluted to a final volume of 10 mL with distilled water. The reduced sugar content of the diluted sample was measured to determine the total sugar content using the 3,5-dinitrosalicylic acid assay [15].

### 2.8. Extraction and Measurement of Dietary Fiber

The detection of dietary fiber was carried out based on a previously reported procedure with minor modifications [16]. First, 1 g of the sample was transferred into a container and added with 15 mL of distilled water. The pH value of the solution was regulated to 7, then added with 0.035 g of α-amylase and incubated at a water bath temperature for 1 h. The pH was then raised to 10, and 0.035 g of alkaline protease was introduced, followed by an additional 1 h incubation. The pH was lowered to 4.5, followed by supplementation with 0.035 g of saccharase for a final 1 h incubation. A centrifuge was used to collect the supernatant and the residual solid. The residual solid after drying was considered as insoluble dietary fiber. Four volumes of 95% anhydrous ethanol were added to the supernatant, and the mixture was allowed to stand overnight. Following centrifugation, the supernatant was removed, and the precipitate was dried at 105 °C to yield soluble dietary fiber.

### 2.9. Extraction and Determination of Free and Bound Phenolics

Phenolics were extracted based on the previous method with minor modifications [17]. First, 1.00 g of quinoa powder was precisely weighed into a 50 mL centrifuge tube and mixed with 10 mL of n-hexane thoroughly. The mixture was allowed to stand overnight. The supernatant was discarded, with the subsequent addition of 10 mL of 80% methanol and ultrasonic extraction at 40 °C for 30 min. The sample was centrifuged at 5000 rpm for 10 min, after which the supernatant was collected, and the extraction was repeated. The supernatants were pooled to obtain the free phenolic fraction. Then, 10 mL of a 2 M NaOH solution was added for the solid residue, and alkaline hydrolysis was performed at 40 °C for 4 h. After hydrolysis, the pH was adjusted to 2 with 6 M HCl, and the solution was boiled in a water bath for 1 h. The sample was centrifuged at 5000 r/min for 10 min, and the supernatant was collected, which represented the bound phenolic fraction. Both the free phenolic supernatant and the bound phenolic supernatant were concentrated to a volume of 1 mL using a rotary evaporator. Storage of the samples was performed at 4 °C for subsequent analysis. The Folin–Ciocalteu assay was employed to quantify the phenolics in the samples [18]. The phenolic content was quantified as gallic acid equivalents (GAEs) in mg GAE/g dry weight (DW).

### 2.10. Determination of Antioxidant Activities of Free Phenolics Extracted from Fermented and Unfermented Quinoa

#### 2.10.1. DPPH Radical Scavenging Ability

The DPPH radical scavenging activity of the samples was determined using a method adapted from a previous study [19]. The samples were formulated with a range of concentrations. A primary DPPH solution was prepared by dissolving 5 mg of DPPH in 10 mL of ethanol and further diluting it to achieve an absorbance of 0.8 ± 0.02 at 517 nm. Then, 50 μL of each sample was combined with 150 μL of the DPPH working solution and allowed to incubate in a dark environment at ambient temperature for 30 min. The absorbance was then determined at 517 nm, and the DPPH radical scavenging ability was computed using Formula (5):

(5) $$DPPH\;radical\;scavenging\;ability\left(\%\right)=\left(\frac{Ac-As}{Ac}\right)\times 100$$

Here, As denotes the absorbance of the sample solutions, and Ac represents the absorbance of the control solution.

#### 2.10.2. ABTS Radical Scavenging Ability

The ABTS radical scavenging ability of the samples was determined using a modified method from a previous publication [20]. Samples were prepared at various concentrations. ABTS radicals were produced by combining 10 mL of a 7.4 mM ABTS solution with 180 μL of a 2.45 mM potassium persulfate solution, followed by allowing the mixture to react in the dark for 16 h. The ABTS radical solution was subsequently diluted with ethanol to achieve an absorbance of 0.8 ± 0.02 at 734 nm. A total of 50 μL of each sample was combined with 150 μL of the ABTS radical cation working solution. The mixture was then incubated in the dark at room temperature for 10 min. Following incubation, absorbance was measured at 734 nm, and the ABTS radical scavenging ability was calculated using Formula (6):

(6) $$ABTS\;radical\;scavenging\;ability\left(\%\right)=\left(\frac{Ac-As}{Ac}\right)\times 100$$

Here, Ac and As are the absorbance of the control solution and sample solution, respectively.

### 2.11. In Vitro Gastrointestinal Digestion and Bioaccessibility

In vitro digestion of fermented and unfermented quinoa samples was performed using a method adapted from a previous study [21]. The simulated oral fluid was formulated by dissolving 0.05625 g of α-amylase in 1 L of a CaCl_2_ solution (1 mM), with the pH adjusted to 6.5. For gastric fluid simulation, 8 g of pepsin was added to 1 L of a CaCl_2_ solution (3 mM), and the mixture was acidified to pH 2.0. The intestinal fluid was prepared by dissolving 4.0 g of pancreatin and 25.0 g of bile salts in 1 L of a NaHCO_3_ (0.1 M) solution, with the pH adjusted to 7.4. Initially, the fermented and unfermented quinoa sample (1.0 g) was dispersed in 9 mL of pH-adjusted distilled water at 6.5 and then incubated with artificial saliva at 37 °C for 5 min. After centrifugation, the pellet was treated with 9 mL of distilled water at pH 2 and incubated with simulated gastric fluid for 2 h. After another round of centrifugation, the pellet was re-suspended in 9 mL of pH-adjusted distilled water at 7.4 and incubated with simulated intestinal fluid for an additional 2 h. The aqueous phase collected following the final centrifugation was then analyzed for phenolic content. The bioaccessibility was calculated using Formula (7):

(7) $$bioaccessibility\left(\%\right)=\left(\frac{ratio\;of\;phenolics\;post-digestion}{ratio\;of\;phenolics\;pre-digestion}\right)\times 100$$

### 2.12. Determination of Saponins

A quantitative analysis of saponins was executed, with minor adjustments, according to the methodology detailed in a previous publication [22]. First, 0.1 g of the samples, both prior to and post-fermentation, were precisely weighed. Then, 2 mL of a 50 mM EDTA solution was introduced into each sample, followed by thorough mixing and then a 30 s vortexing process. Subsequently, the foam-generated height was quantified using a calibrated ruler.

### 2.13. Strain Screening Using PCA

PCA was utilized to develop an evaluation model for the quinoa fermentation process, enabling the derivation of comprehensive scores for all samples [23].

By examining the eigenvalues and loadings of the initial variables, the principal component Yi can be delineated as follows (8):

(8) $$F_{i}=a_{i1}X_{1}+a_{i2}X_{2}+a_{i3}X_{3}+a_{i4}X_{4}+\cdots +a_{in}X_{n}$$

Here, a_ij_ represents the component coefficient associated with the eigenvalue.

Subsequently, the score for each principal component was weighted by its corresponding contribution to the variance, denoted as b, to yield the overall scoring model for the fermented quinoa samples.

(9) $$F=b_{1}F_{1}+b_{2}F_{2}+b_{3}F_{3}+b_{4}F_{4}+\cdots +b_{n}F_{n}$$

### 2.14. Preparation and Analysis of Quinoa Fermented Milk

The quinoa was thoroughly cleaned by repeated rinsing until it was devoid of any foam. After soaking for 6 h, water was incorporated at a ratio of 1:15 (weight of quinoa to volume of water), and the mixture was subjected to high-speed blending until a smooth, and grain-free consistency was achieved. Then, 14% quinoa pulp and 2% sucrose were added to 100 g of milk and blended at a high speed for 3 min. Subsequently, the Maillard Browning reaction was facilitated by heating the mixture to a temperature of 121 °C for 15 min. After this reaction, the brown milk was cooled, and 3% of the selected optimal strain (*MS2*) was inoculated into the quinoa milk, and a controlled fermentation process was initiated at 37 °C for 5 h. Fermentation was stopped at 4 °C, and after 24 h of refrigeration, brown quinoa fermented milk (WS) was obtained. And unfermented quinoa milk homogenate (CK) represented the control group. The physicochemical parameters, including the pH, titratable acidity, water holding capacity (WHC), suspension stability, and color, of the fermented milk were evaluated on days 1, 7, 14, and 21.

#### 2.14.1. Determination of pH and Titratable Acidity (TA)

A pH meter (a-AB33PH-FZH, Ohaus Instrument Co., Ltd, Changzhou, China) was employed to measure the pH value of the quinoa fermented milk. Before measurement, the fermented milk sample was thoroughly mixed, and a pH electrode was inserted. Then, 10 g (weighed to 0.001 g accuracy) of the homogenized fermented milk were transferred to a 100 mL beaker. To this, 20 mL of pre-boiled and cooled water was added, and the mixture was vigorously stirred until homogeneous. Subsequently, 2.0 mL of a phenolphthalein indicator was added, and the fermented milk was titrated with a 0.1 M NaOH standard solution to determine its acidity [24]. The calculation formula was as follows (10):

(10) $$TA=c\times \left(V-V_{0}\right)\times \frac{100}{m\times 0.1}$$

where TA represents sample acidity (°T), c denotes the molar concentration of the NaOH standard solution (M), V indicates the volume of sodium hydroxide consumed during titration (mL), V_0_ represents the volume of a sodium hydroxide standard solution consumed in the blank experiment (mL), and m signifies the mass of the sample (g).

#### 2.14.2. Rheological Analysis of Quinoa Fermented Milk

The rheological behavior of quinoa fermented milk was evaluated based on a previously reported method [25]. The samples were subjected to rheological analysis at room temperature using the MCR102 rheometer (Anton Paar Co., Ltd., Graz, Austria). The shear rate was systematically varied from 0.01 to 100 S^−1^, and 50 data points were recorded to plot the apparent viscosity against the shear rate. Dynamic rheological properties were assessed at 25 °C with a 0.5% shear strain over 10 min, with the frequency ranging from 0.1 to 10 Hz, and changes in the G′ and G″ moduli were monitored. In the shear stress–shear rate analysis, the shear rate was incrementally adjusted from 0.01 to 100 S^−1^ and then decremented back to 0.01 S^−1^, capturing 50 data points in each direction. The curve was constructed with shear rate on the horizontal axis and shear stress on the vertical axis.

#### 2.14.3. Sensory Evaluation Measurements

Five trained sensory evaluators were selected to constitute a sensory evaluation panel. Sensory assessment criteria and procedural guidelines were clearly established, and quinoa fermented milk was evaluated across five sensory attributes—color, taste, aroma, texture, and overall acceptability—with 20 points maximum per attribute and a total of 100 points. Scores were calculated as the mean value, with detailed criteria provided in Table S1.

#### 2.14.4. Electronic Tongue Determination

A specific quantity of the sample was weighed and diluted by five folds with constant stirring to ensure thorough and even mixing of water with the fermented milk sample. After centrifugation at 3000 rpm for 10 min, the supernatant was decanted and tested with the electronic tongue SA402B (Insent Inc., Atsugi-chi, Japan).

#### 2.14.5. GC-IMS Measurement

A gas chromatography (GC)–ion mobility spectrometry (IMS) instrument (FlavourSpec1H1-00053 from G.A.S., Dortmund, Germany) was employed to characterize the aroma profile of quinoa fermented milk. The analytical method was modified based on a previously reported procedure [25]. First, 2 g of the sample was sealed in a 20 mL headspace vial and thermally equilibrated with shaking at 60 °C for 15 min. Subsequently, 500 μL of the headspace gas was withdrawn for detection, with the injection needle temperature maintained at 85 °C. Separation was carried out using an IMS chromatographic column (WAX; 15 m; ID: 0.53 mm). High-purity nitrogen (99.999%) served as both the carrier and drift gases. GC-IMS test conditions were configured as follows: the GC column temperature was set to 60 °C, and the drift tube temperature was maintained at 45 °C. The carrier gas flow rate was programmatically adjusted to 2 mL/min for the initial 2 min, increased to 10 mL/min from 2 to 10 min, further ramped to 100 mL/min between 10 and 20 min, and finally set to 150 mL/min from 30 to 35 min. The drift gas flow rate was kept constant at 150 mL/min throughout the analysis. Each sample was analyzed in triplicate.

#### 2.14.6. Determination of Viable Bacterial Count and Color

The viable counts of *L. casei*, *L. fermentum* and *L. rhamnosus* in quinoa fermented milk were determined using the plate count method [26]. One milliliter of the diluted sample was placed into a petri dish, and an MRS medium was added. After solidification, the cultures were incubated at 37 °C for 72 h. The colonies were counted after incubation, and the results are expressed as CFU/mL. The color of quinoa fermented milk was measured using the method outlined in Section 2.4 after 1, 7, 14, and 21 days of storage.

#### 2.14.7. Determination of WHC and Suspension Stability

The WHC of quinoa fermented milk was determined using the centrifugation method after 1, 7, 14, and 21 days of storage [27]. Twenty grams of fermented milk was placed into a 50 mL test tube and centrifuged at 5000 rpm for 10 min. The supernatant was collected and weighed. The WHC was calculated using Formula (11):

(11) $$WHC\;\left(\%\right)=\frac{M_{1}-M_{2}}{M_{1}}\times 100$$

Here, M_1_ denotes the initial mass of the fermented milk sample, and M_2_ denotes the mass of the supernatant after centrifugation.

Quinoa fermented milk was measured for its suspension stability [28] after 1, 7, 14, and 21 days of storage. Briefly, after dilution of the fermented milk sample by 50 folds with distilled water, the absorbance value at 540 nm was measured and recorded as A_1_. Then, 15 mL of fermented milk was centrifuged at 4500 rpm for 15 min, and the absorbance value of the upper emulsion at 540 nm was measured and recorded as A_2_. The suspension stability R of the quinoa fermented milk was calculated according to Formula (12):

(12) $$R=\frac{A_{2}}{A_{1}}$$

### 2.15. Statistical Analysis

All experiments were performed in triplicate, and data are presented as the mean (n = 3) ± standard deviation (SD). Statistical analysis was conducted using Duncan’s test and one-way ANOVA via SPSS 26.0 (IBM, Armonk, NY, USA), with statistical significance set at *p* < 0.05. PCA for strain screening was performed using the same software.

## 3. Results and Discussion

### 3.1. Impact of Fermentation Using Different Strains on Quinoa Color

Color is one of the most important characteristics for assessing the edibility and quality of a food product. Table 2 presents the L*, a *, and b * values of quinoa before and after fermentation. The L*, a*, and b* values of unfermented quinoa were determined to be 68.63 ± 0.17, 3.67 ± 0.15, and 12.32 ± 0.62, respectively. Subsequent fermentation with various strains resulted in a significant decrease in L* value and a significant increase in a* value of quinoa. Notably, fermentation with strain *LF* led to the most significant reduction in L* value, while fermentation with strain *MS4* resulted in the most dramatic increase in a* value, which reached 6.37 ± 0.22. However, fermentation seemed to have a minimal impact on the b* value of quinoa, regardless of the strains. Collectively, fermentation significantly reduced the total color of quinoa, which may be due to the enzymatic activity of microorganisms during fermentation, such as β-glucosidase, which can hydrolyze natural pigments, like flavonoids [29]. Additionally, the non-enzymatic browning reaction during fermentation may also contribute to the color changes observed in fermented quinoa.

### 3.2. Effects of Fermentation Using Various Strains on Nutrient Content of Quinoa

**Figure 1:**
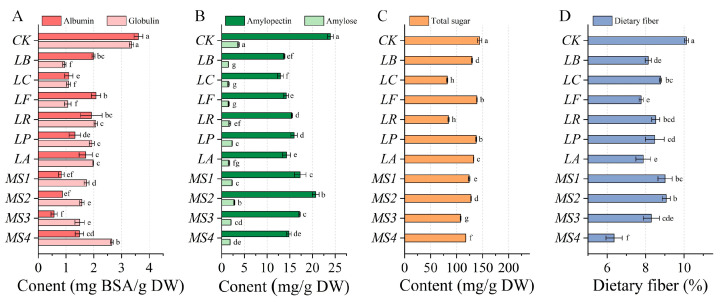
Effects of fermentation with various LAB strains on the contents of protein (**A**), starch (**B**), total sugar (**C**), and dietary fiber (**D**) of quinoa. Note: *CK*, unfermented quinoa (control group). *LB*, *LC*, *LF*, *LR*, *LP*, *LA*, *MS1*, *MS2*, *MS3*, and *MS4* represent fermented quinoa (different treatment groups). Different lowercase letters denote statistically significant differences (*p* < 0.05) between samples.

Variations in the protein content of quinoa before and after fermentation are depicted in Figure 1A. Prior to fermentation, the contents of albumin and globulin in quinoa were 3.61 ± 0.16 and 3.36 ± 0.08 mg BSA/g DW, respectively. Fermentation with various LAB strains significantly reduced the content of both albumin and globulin in quinoa. Notably, the most substantial decrease in albumin content was observed after fermentation with strain *MS3* (by 84.1%), while the most pronounced decrease in globulin content was found after fermentation with strain *LB* (by 71.9%). These decreases can be due to the utilization of proteins from quinoa by the microorganisms as a nitrogen source to sustain their growth and proliferation during the fermentation process. Additionally, the proteases produced by LAB strains can hydrolyze the proteins in quinoa, generating peptides that are further degraded into free amino acids, and yield intermediate compounds during aromatic product synthesis [13,30].

Changes in the starch content of quinoa before and after fermentation with different LAB strains are illustrated in Figure 1B. Unfermented quinoa contained 23.97 ± 0.64 mg/g of amylopectin and 3.72 ± 0.19 mg/g of amylose. Fermentation decreased the amylopectin and amylose content in quinoa by 13.58–45.99% and 24.62–60.18%, respectively. Notably, fermentation with the *LB*, *LC*, and *LF* strains resulted in the most pronounced decrease in both amylopectin and amylose. These findings are consistent with previous studies demonstrating that LAB fermentation can significantly reduce the starch content in cereals and pseudocereals. For example, a study reported a 20–30% reduction in starch content in sourdough wheat after fermentation with *Lactobacillus plantarum*, attributed to the enzymatic activity of amylases and glycosidases produced by LAB [31]. Similarly, Ren et al. observed a 15–25% decrease in starch content in fermented millet, highlighting the role of fermentation in modifying the composition of carbohydrates [32]. In addition, the total sugar content of unfermented quinoa was 144.25 ± 4.02 mg/g, which was also decreased to varying degrees after fermentation (Figure 1C). Fermentation with the *LC* strain led to the most substantial decrease in the total sugar content (43.15%); by contrast, fermentation with the *LP* strain only decreased the total sugar content by 4.56%. It has been noted that LAB strains differ in their ability to metabolize sugars, depending on their enzymatic profiles [33]. Strains such as *LC* are known to produce higher levels of glycosidases, which may explain their greater efficiency in sugar utilization compared to *LP*. Similarly, fermentation significantly decreased the dietary fiber content in quinoa (Figure 1D), among which the mixed-strain *MS4* resulted in the most significant reduction (by 37.24%). Previous research has reported a 25–35% reduction in the dietary fiber content in sprouted quinoa following fermentation with LAB [34]. This decrease was attributed to the activity of cellulases and hemicellulases produced by LAB during the fermentation process. In the present study, the pronounced effect observed with the mixed-strain *MS4* may be attributed to the synergistic action of multiple LAB strains. Studies have indicated that mixed cultures of LAB often exhibit enhanced enzymatic activity compared to single-strain cultures [35], providing a mechanistic basis for the improved performance of multi-strain combinations in fermentation applications. LAB strains can utilize starch and sugars in quinoa as carbon sources for their metabolic activities and produce a variety of enzymes, including amylases, non-starch carbohydrate hydrolases, and cellulases, which are capable of breaking down starch, sugars, and dietary fiber, leading to decreases in their contents in quinoa [36,37]. In summary, fermentation with various LAB strains may induce notable differences in the nutrient composition of quinoa.

### 3.3. Effects of Fermentation on Phenolic Content and Antioxidant Ability of Quinoa

**Figure 2:**
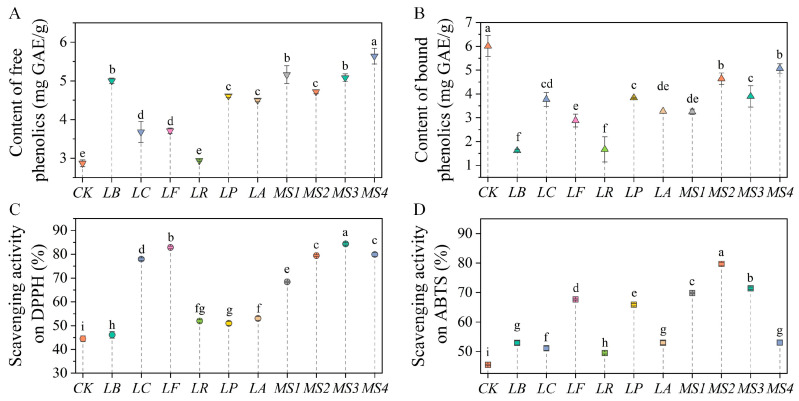
Effects of fermentation using various LAB strains on the content of free phenolics (**A**) and bound phenolics (**B**), DPPH (**C**), and ABTS (**D**) radical scavenging in quinoa. Note: *CK*, unfermented quinoa (control group). *LB*, *LC*, *LF*, *LR*, *LP*, *LA*, *MS1*, *MS2*, *MS3*, and *MS4* represent fermented quinoa (different treatment groups). Different lowercase letters denote statistically significant differences (*p* < 0.05) between samples.

Variations in the phenolic content of quinoa before and after fermentation with various LAB strains are depicted in Figure 2A,B. The level of free and bound phenolics in unfermented quinoa were 2.87 ± 0.09 and 6.01 ± 0.44 mg GAE/g DW, respectively. After fermentation with various LAB strains, the content of free phenolics in quinoa increased by 2.26–49.10%, with the mixed-strain *MS4* achieving the highest content (5.64 ± 0.2 mg GAE/g DW) after fermentation. Conversely, the content of bound phenolics in quinoa showed a significant decrease after fermentation. This decrease is likely due to the metabolic activities of fermenting microorganisms. These organisms secrete enzymes, including β-glucosidase, decarboxylases, hydrolases, and reductases, which catalyze the cleavage of chemical bonds linking phenolic compounds to cell wall components. This enzymatic action releases bound phenolics as free forms. Moreover, microbial metabolism causes cell wall disruption, thereby promoting the synthesis of bioactive compounds [38]. Fermentation-induced acidification further accelerates the hydrolysis of complex phenolic compounds, converting glycosides into phenolic acids and increasing the overall free phenolic content [8]. However, the changes in phenolic content after fermentation with different LAB strains exhibited certain variations. A study applying *L. rhamnosus* SP1 and *L. plantarum* T6B10 to ferment quinoa and prepare yogurt-like beverages found that despite significant increases in the total phenolic content in each fermented beverage, there were great variations in the extent of increase [39], which may be closely related to the metabolic differences among different strains.

In unfermented quinoa samples, free phenolics exhibited 44.51% and 45.47% scavenging capacities against DPPH and ABTS radicals, respectively. LAB fermentation was shown to significantly enhance the antioxidant potential of free phenolics in quinoa (Figure 2C,D). A previous study showed that the IC_50_ values of quinoa seeds before and after fermentation with *Lactobacillus casei* are 6.65 and 3.43 mg/mL, respectively, indicating that fermentation increases the antioxidant capacity of quinoa [11]. There were considerable variations in the scavenging capabilities against DPPH and ABTS radicals in quinoa fermented with different LAB strains. For instance, after fermentation with the mixed-strain *MS2*, the scavenging rate of free phenolics against the DPPH and ABTS radicals increased by 34.92% and 34.24%, respectively. In contrast, fermentation with the *LR* strain resulted in only 7.46% and 4.01% increases in the scavenging rate against the DPPH and ABTS radicals, respectively. This variation can be attributed to the concentration and chemical form of antioxidant components in plant-based foods [8]. Fermentation can significantly alter the level and type of phenolic compounds, thereby influencing the overall antioxidant potential of a substance. Moreover, numerous species of LAB are equipped with both enzymatic and non-enzymatic systems, which contribute to their antioxidant capabilities [40]. However, the varying capacities of LAB strains to transform phenolic compounds into bioactive derivatives via phenolic acid decarboxylase, glycosidase, or esterase can cause substantial disparities in the antioxidant activity of the final products [41].

### 3.4. Effects of Fermentation on Bioaccessibility of Phenolics in Quinoa

**Figure 3:**
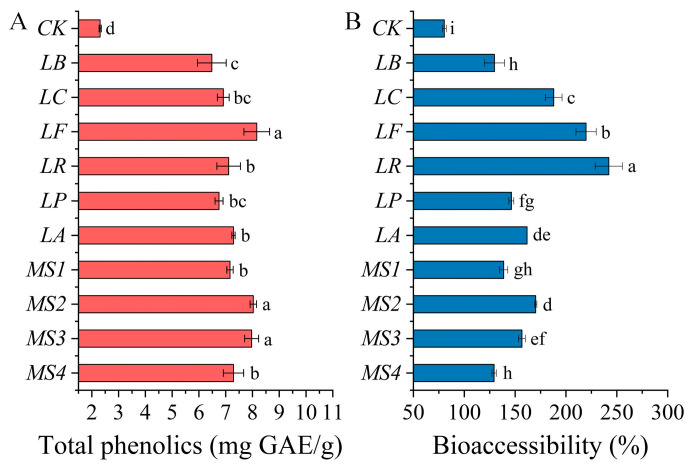
Total phenolic content (**A**) and bioaccessibility (**B**) in quinoa after simulated digestion in vitro. Note: *CK*, unfermented quinoa (control group). *LB*, *LC*, *LF*, *LR*, *LP*, *LA*, *MS1*, *MS2*, *MS3*, and *MS4* represent fermented quinoa (different treatment groups). Different lowercase letters denote statistically significant differences (*p* < 0.05) between samples.

The release and bioaccessibility of phenolics from different samples after in vitro gastrointestinal digestion are depicted in Figure 3A,B. Following simulated gastrointestinal digestion, the release rate of phenolics from unfermented quinoa was 2.31 mg GAE/g DW, with bioavailability reaching up to 80.35%. Fermentation with various LAB strains significantly enhanced the phenolic content and bioavailability in quinoa by 1.80–2.53 folds and 0.61–2.01 folds, respectively. It has been demonstrated that digestive enzymes in the stomach and intestines can facilitate the release of phenolic compounds from grains, as the phenolic compounds in grains are predominantly bound to macromolecules, such as proteins, starch, and polysaccharides, through glycosidic and ester linkages. Under the influence of enzymes, such as α-amylase, gastric acid, pepsin (capable of hydrolyzing polysaccharide carboxyl groups), and pancreatic enzymes (capable of hydrolyzing starch glycosidic bonds and protein ester bonds), these phenolic compounds can be gradually hydrolyzed and released [42]. Furthermore, the structure of phenolics in grains may be changed during fermentation, which can affect their solubility and transmembrane transport capacity during the simulated digestion process, thereby significantly increasing the bioavailability of phenolics in fermented quinoa [43].

### 3.5. Effects of Fermentation Using Various Strains on Saponin Content in Quinoa

**Figure 4:**
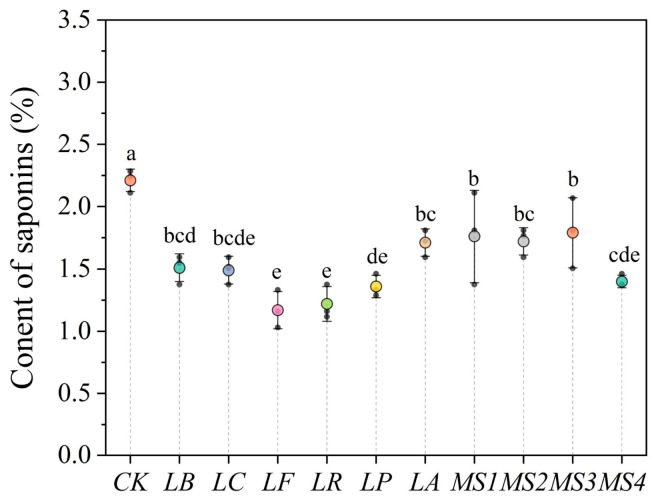
Effects of fermentation using various LAB strains on the saponin content of quinoa. Note: *CK*, unfermented quinoa (control group). *LB*, *LC*, *LF*, *LR*, *LP*, *LA*, *MS1*, *MS2*, *MS3*, and *MS4* represent fermented quinoa (different treatment groups). Different lowercase letters denote statistically significant differences (*p* < 0.05) between samples.

Saponins, characterized by their bitter taste, form a complex with minerals like zinc and iron. This interaction impairs product sensory quality and hinders the body’s absorption of these minerals [1]. Previous research has demonstrated that fermentation with *Lactobacillus plantarum* can reduce the content of antinutritional saponins in quinoa from 2.84% to 2.04% [10]. Our results also indicate that fermentation of quinoa with different LAB strains can significantly decrease the saponin content (Figure 4). Before fermentation, the saponin content in quinoa was 2.21%, and fermentation reduced the saponin content to 1.17–1.79%. Among different strains, *LF* resulted in the most significant reduction in saponin content in quinoa. The *LR*, *LP*, *LC*, and *MS4* strains showed no significant differences from LF and also affected the reduction in saponin content. Thus, fermentation can diminish the antinutritive factors in quinoa and can result in acceptable sensory characteristics.

### 3.6. Selection of Better Starter Culture for Quinoa Fermentation Based on Principal Component Analysis

**Figure 5:**
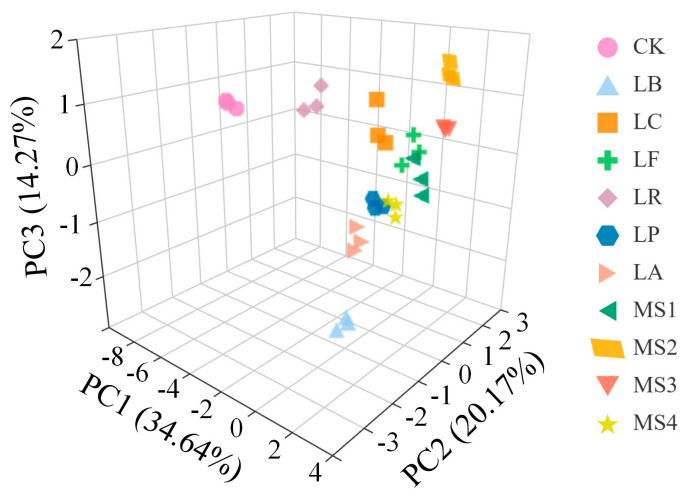
Principal component analysis (PCA) score plot of fermented quinoa with different LAB strains. Note: *CK*, unfermented quinoa (control group). *LB*, *LC*, *LF*, *LR*, *LP*, *LA*, *MS1*, *MS2*, *MS3*, and *MS4* represent fermented quinoa (different treatment groups).

In this study, SPSS 26.0 was utilized to perform PCA on various indices of quinoa fermented by different LAB strains, including free phenolics, bound phenolics, antioxidant capacity, saponins, and bioavailability. The results indicate that the cumulative contribution rate of the first three principal components (PCs) reached 69.08% (Figure 5). Since each PC represents a linear combination of the initial variables, the influence of each variable on the PC can be represented by the loadings, with a higher absolute value of loadings indicating greater influence.

Different PCs reflect the nutritional, functional, and physicochemical characteristics of fermented quinoa. The amylose and amylopectin content and antioxidant properties were the main feature vectors affecting PC1; free phenolic content, phenolic release after simulated digestion, and their bioavailability were the main feature vectors affecting PC2; while dietary fiber and saponin content and color were the main feature vectors affecting PC3. Using the component score coefficient matrix (Table S2) and eigenvalues (Table S3), the scoring model for the principal components is presented as follows:

(13) $$F_{1}=0.26X_{1}+0.31X_{2}+0.27X_{3}+0.25X_{4}+0.39X_{5}-0.36X_{6}+0.07X_{7}+0.10X_{8}\;+0.38X_{9}+0.38X_{10}+0.13X_{11}-0.12X_{12}+0.23X_{13}-0.17X_{14}$$

(14) $$F_{2}=-0.43X_{1}-0.15X_{2}-0.15X_{3}+0.20X_{4}+0.19X_{5}+0.04X_{6}-0.19X_{7}+0.24X_{8}\;+0.04X_{9}+0.14X_{10}-0.17X_{11}-0.39X_{12}+0.35X_{13}+0.53X_{14}$$

(15) $$F_{3}=0.12X_{1}+0.26X_{2}-0.25X_{3}+0.31X_{4}-0.08X_{5}+0.21X_{6}+0.33X_{7}-0.59X_{8}\;-0.14X_{9}-0.12X_{10}+0.14X_{11}-0.29X_{12}+0.33X_{13}+0.05X_{14}$$

(16) $$F_{4}=0.11X_{1}-0.31X_{2}-0.09X_{3}-0.36X_{4}+0.20X_{5}+0.37X_{6}-0.10X_{7}+0.02X_{8}\;+0.10X_{9}+0.16X_{10}+0.67X_{11}-0.27X_{12}+0.03X_{13}-0.09X_{14}$$

(17) $$F_{5}=-0.14X_{1}+0.00X_{2}-0.10X_{3}-0.34X_{4}-0.17X_{5}+0.02X_{6}+0.69X_{7}+0.03X_{8}\;+0.36X_{9}+0.30X_{10}-0.20X_{11}-0.16X_{12}-0.20X_{13}+0.13X_{14}$$

where F_1_–F_5_ are the scores of the principal components, and X_1_–X_14_ denote the standardized values of free phenolics, bound phenolics, saponins, the scavenging DPPH free radical, the scavenging ABTS free radical, albumin, globulin, dietary fiber, amylose, amylopectin, total sugar, color, total phenolics after digestion, and bioaccessbility, respectively. Utilizing the weights of each principal component, the following comprehensive score function was established:

(18) $$F=0.346F_{1}+0.202F_{2}+0.143F_{3}+0.106F_{4}+0.090F_{5}$$

The results are shown in Table S4. The highest overall score was obtained for the mixed-strain *MS2*. Hence, *MS2* was selected as a fermenting agent to prepare quinoa fermented milk, and the sensory characteristics and storage stability of the prepared fermented milk were investigated.

### 3.7. Physical and Chemical Indicators of Quinoa Fermented Milk

#### 3.7.1. Variations in pH and TA During Fermentation Stage

**Figure 6:**
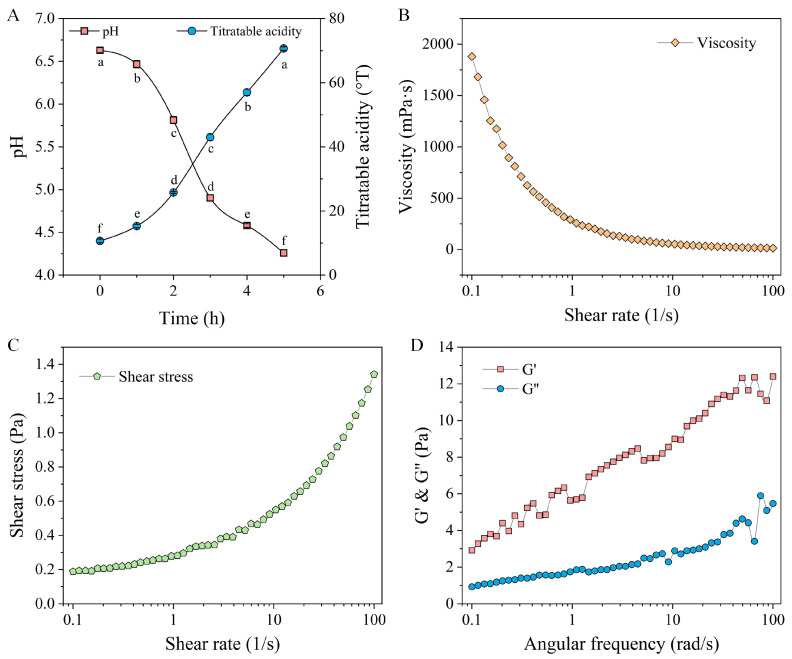
Changes in pH and TA during fermentation (**A**); viscosity and shear rate (**B**); shear stress and shear rate (**C**); G′ and G″ (**D**) of quinoa fermented milk.

Acidity is a key parameter in milk fermentation, which is typically monitored using pH and TA to determine the endpoint of milk fermentation [44]. Figure 6A illustrates the fluctuations in pH and TA throughout the fermentation of quinoa fermented milk.

The initial pH of the sample was 6.63 ± 0.03. After one hour of fermentation, the pH of the quinoa fermented milk gradually decreased, while TA exhibited a progressive increase. From the second to the fifth hour of fermentation, there was a significant reduction in pH in the quinoa fermented milk. After five hours of fermentation, the TA of the sample reached 70.67 °T. The increase in acidity can be attributed to the generation of organic acids, particularly lactic acid, which possess certain preservative and antimicrobial characteristics in fermented foods [45]. The pH value of the sample at the endpoint of fermentation was 4.07, which is a typical value for fermented dairy products and is commonly regarded as an indicator of high-quality product standards [46].

#### 3.7.2. Rheological Properties

Viscosity quantifies a liquid’s resistance to flow under shear stress, offering insights into the characteristics of dairy products [47]. Rheological properties can indicate how viscosity changes with varying shear rates. The relationship between the viscosity and the shear rate of the samples is illustrated in Figure 6B. The results demonstrate that as the shear rate increased, the samples’ apparent viscosity dropped sharply and subsequently leveled off, suggesting significant pseudo-plastic behavior and the shear-thinning effect typical of non-Newtonian fluids [48]. At low shear rates, there was minimal disruption of the internal gel network structure of the samples. Subsequently, with an increasing shear rate, the disruption of the internal gel network structure gradually intensified until the occurrence of complete disruption. As a result, electrostatic and hydrophobic interactions were weakened, ultimately leading to a shear-thinning phenomenon [49]. Simultaneously, the shear stress of the samples increased with an increasing shear rate (Figure 6C). To delve deeper into the internal structure of the fermented milk, dynamic rheological properties were assessed, specifically, the storage modulus (G′) and the loss modulus (G″). The G′ and G″ values for the fermented milk samples exhibited an increasing trend with an increase in frequency (Figure 6D). Furthermore, G′ remained higher than G″ throughout the measurements, confirming the semi-solid weak gel structure of the fermented milk sample, as previously reported [50]. The absence of a crossover between G′ and G″ further implied a predominantly solid-like architecture, contributing to its superior storage stability [51].

#### 3.7.3. Sensory Profiling

**Figure 7:**
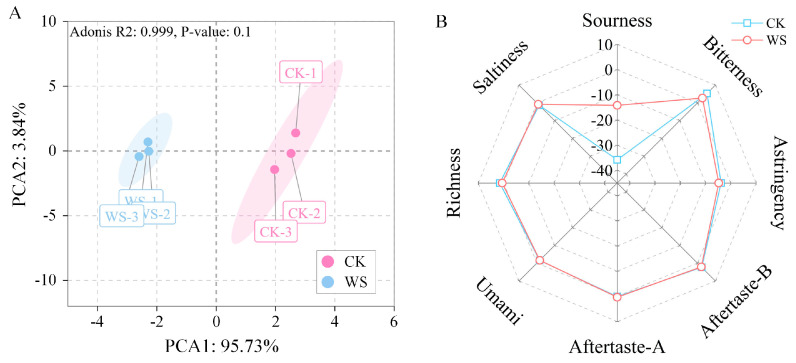
PCA plots (**A**) and radar plots (**B**) of electronic tongue analysis of quinoa fermented milk. Note: *CK*, unfermented quinoa homogenate; WS, quinoa fermented milk.

An electronic tongue, a bionic device for taste analysis, can objectively quantify taste changes in food. It should be emphasized that the descriptors “bitterness” and “astringency” do not necessarily indicate an intense bitter flavor in the fermented milk. Rather, these descriptors characterize the complex and well-rounded mouthfeel of the fermented milk. Figure 7A presents the PCA plot of the detection and discrimination results between the quinoa fermented milk (WS) and unfermented quinoa homogenate (CK) obtained by the electronic tongue. PC1 (95.73%) and PC2 (3.84%) together accounted for 99.57% of the total variance. There was a distinct separation between the two groups, indicating significant differences in their taste perception and successful preparation of quinoa fermented milk with a unique taste profile (Figure 7B). The average sensory score of the quinoa fermented milk prepared using MS2 was 93.2 points, where the sensory scores for color, aroma, texture, flavor, and overall acceptability all exceeded 15 points (Figure S1). These results indicate that the prepared quinoa fermented milk had a light brown color and a glossy appearance, a harmonious aroma, a roasted fragrance and natural scent, a delicate and smooth mouthfeel, a uniform texture, and no signs of layering.

**Figure 8:**
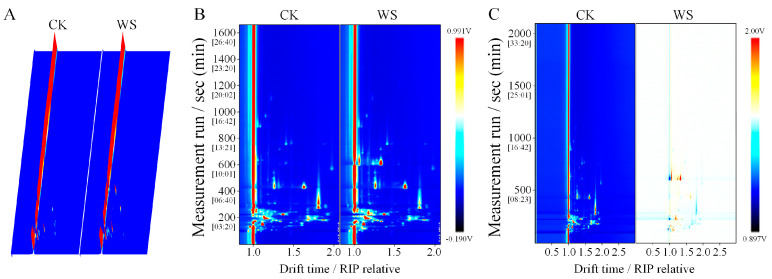
GC-IMS spectra of quinoa fermented milk. (**A**) Three-dimensional spectrum plot. (**B**) Two-dimensional top view. (**C**) Two-dimensional difference map. Note: *CK*, unfermented quinoa homogenate; WS, quinoa fermented milk.

Gas chromatography–ion mobility spectrometry (GC-IMS) was employed to analyze the volatile compounds in quinoa fermented milk. Figure 8A illustrates the three-dimensional ion mobility spectra of different samples, which can visually represent the volatile organic compounds (VOCs) in the samples. Due to the inconvenience of observations, a top-down view was utilized for differential comparisons. Figure 8B presents the ion mobility spectra of VOCs in the samples, with the red vertical line at the abscissa of 1.0 representing the reaction ion peak (RIP peak). The ordinate represents the gas chromatography retention time (s), and the abscissa represents the ion mobility time. For each point flanking the RIP peak, it represents a distinct type of volatile organic compound (VOC). As shown in Figure 8C, GC-IMS effectively separated the VOCs in WS and CK. To more intuitively reflect the differences in VOCs between WS and CK, a two-dimensional differential spectral map was generated. The spectrum of the CK group was selected as a reference, from which the spectrum of the WS sample was subtracted. After the subtraction, the background would be white if the VOCs of the two groups were identical, red if the VOC concentration was higher, and blue if the VOC concentration was lower in WS compared with that in CK (Figure 8C). The WS group contained a more diverse array of VOCs, with different compounds having distinct flavor characteristics.

**Figure 9:**
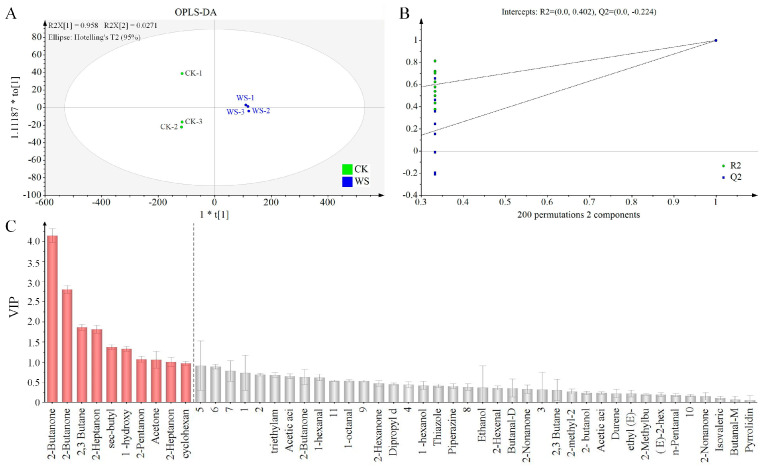
Multivariate statistical analysis of volatile compounds in different samples. (**A**) Scatter maps of OPLS-DA. (**B**) Cross-validation test. (**C**) VIP distributions.

Table S5 lists the composition of the VOCs in the samples. The VOCs labeled as M and D signify their monomeric and dimeric forms, respectively, while Arabic numerals indicate unidentified compounds. A total of 48 VOCs were detected in the samples, among which 37 were successfully identified, including eight aldehydes, five esters, 13 ketones, three alcohols, one acid, and seven others. OPLS-DA was conducted on the VOCs of WS and CK to test their differences (Figure 9A). The analysis indicated that the cumulative contribution rate of PC1 and PC2 was 98.51%, with R^2^X = 0.985, R^2^Y = 0.999, and Q^2^ = 0.998. R^2^ and Q^2^ values greater than 0.9 suggested that the model has accurate explanatory and predictive capabilities. Figure 9B displays the validation results of the OPLS-DA model after 200 permutations, where R^2^ intersects with the *y*-axis at (0, 0.402), and Q^2^ intersects with the *y*-axis at (0, −0.224), suggesting that the model possesses good predictive ability and effectively captures the flavor profiles of the samples. The variable importance in projection (VIP) was employed to evaluate the contribution of each variable in the orthogonal partial least squares discriminant analysis (OPLS-DA). A total of 10 VOCs with VIP values exceeding the threshold of 1 were identified, including eight ketones, one ester, and one other class of VOCs (Figure 9C).

**Figure 10:**
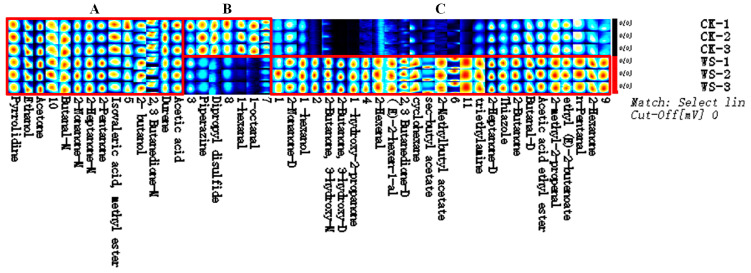
The volatile components’ fingerprint of quinoa fermented milk. Note: *CK*, unfermented quinoa homogenate; WS, quinoa fermented milk.

To further elucidate the characteristics of the VOCs in the samples, retrograde sorting of the identified characteristic peak regions was performed based on peak selection criteria, resulting in the construction of flavor fingerprint spectra of the samples, as shown in Figure 10. In this figure, each row corresponds to all the signal peaks extracted from an individual sample, while each column represents the signal peaks of the same VOC across different samples. It has been well established that the flavor of fermented milk is primarily generated through enzymatic and chemical reactions during the fermentation process, including lipid oxidation, carbohydrate fermentation, amino acid catabolism, and microbial metabolism. The VOCs marked in region C of the figure are those with higher concentrations in the fermented milk samples. Specifically, 2-butanone, 3-hydroxy, and 2,3-butanedione-D contribute to a creamy aroma; 1-hydroxy-2-propanone has a caramel-like scent; 2-hexenal brings about a sweet almond flavor; (E)-2-hexen-1-al conveys a fruity and green leafy aroma; cyclohexane has a mild sweet taste; 2-methylbutyl acetate has a fruity and sweet fragrance; acetic acid ethyl ester, 2-hexanone, and sec-butyl acetate possess fruity scents; butanal-D has a fruity and green leaf odor; 2-nonanone-D presents a fruity and floral scent; 1-hexanol has a green, fruity, and fatty aroma; 2-methyl-2-propenal has a hyacinth-like odor; ethyl (E)-2-butenoate confers a burnt and fruity fragrance; and n-pentanal has the smell of fermented bread and berries. In general, the quinoa fermented milk prepared in this study exhibits favorable flavor characteristics.

#### 3.7.4. Stability Evaluation During Storage

**Figure 11:**
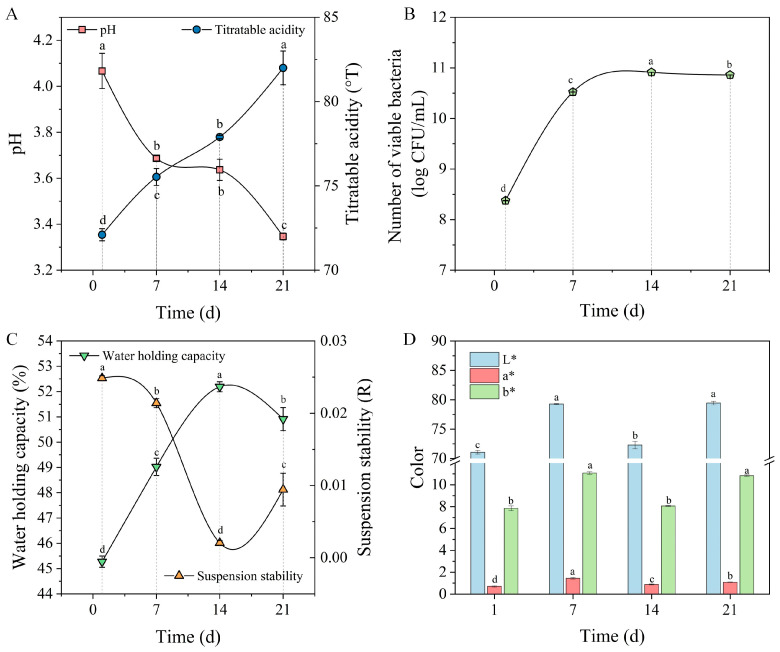
Assessment of physicochemical parameters during quinoa fermented milk storage. (**A**) Variations in pH and titratable acidity. (**B**) Changes in the viable bacteria count. (**C**) Changes in water-holding capacity and suspension stability. (**D**) Changes in lightness (L*), greenness/redness (a*), and blueness/yellowness (b*). Different lowercase letters denote statistically significant differences (*p* < 0.05) between samples.

During storage, variations in pH and TA can adversely affect a product’s quality, thereby reducing its shelf life [52]. Therefore, it is crucial to maintain appropriate acidity in fermented milk during storage. The pH and TA of the prepared quinoa fermented milk stored for 1, 7, 14, and 21 days are depicted in Figure 11A. Over the storage period, although the pH of the fermented milk exhibited a decreasing trend, TA showed an increasing trend and remained within the range of 72.1–82.0 °T. Post-acidification in quinoa fermented milk during storage may be associated with the continuous metabolic activities of LAB under low-temperature conditions. Studies have shown that LAB continues to utilize residual sugars to produce lactic acid during storage, leading to a further decrease in pH [35]. Additionally, other organic acids present in quinoa yogurt, such as acetic acid and succinic acid, may also contribute to the pH decline [53]. In traditional yogurt, pH values typically decrease during storage as well, though at smaller amplitudes, generally ranging between 3.8 and 4.8 [54]. This discrepancy may be attributed to differences in LAB species and their metabolic characteristics. For example, certain LAB strains, such as Lactobacillus plantarum and Lactobacillus rhamnosus, exhibit higher metabolic activity in quinoa-based matrices, thereby accelerating post-acidification [35]. Generally, TA values between 70 and 110 °T are considered acceptable to consumers [55]. Therefore, the prepared quinoa fermented milk could maintain satisfactory acidity during storage.

The viability of bacteria serves as a key indicator for evaluating the effectiveness of probiotic functional foods. To ensure product quality, the viable bacterial count should remain within the range of 6–8 log CFU/mL during the entire shelf life [56]. The viable bacteria count in quinoa fermented milk during storage is illustrated in Figure 11B. At the start of storage, the viable bacteria count in the fermented milk was 8.38 log CFU/mL. With the passage of storage time, the viable bacteria count in the fermented milk significantly increased, reaching 10.91 log CFU/mL by the 14th day. The increase in viable LAB counts during the storage of quinoa fermented milk is primarily attributed to several interconnected factors. First, quinoa is rich in oligosaccharides and polysaccharides that act as prebiotics, providing a sustainable carbon source for LAB [1]. During the fermentation process, milk proteins and lactose are hydrolyzed into peptides, amino acids, and simple sugars, which serve as readily available nutrients for bacterial growth. Although the metabolic activity of microorganisms is reduced at 4 °C storage, it does not cease entirely. Under these conditions, LAB can utilize the available carbon and nitrogen sources derived from quinoa and milk to maintain slow but continuous fermentation. This process generates metabolic byproducts, like lactic acid, which not only lower the pH but also create an acidic environment conducive to LAB survival, thereby supporting sustained growth and reproduction during storage [35]. However, a subsequent decline in the viable bacteria count was observed thereafter. Upon completion of the storage period, the viable bacteria count in quinoa fermented milk was still above 10 log CFU/mL, complying with the minimum viable bacteria count standard established by the Food and Agriculture Organization (FAO) and the World Health Organization (WHO) for probiotic foods [52].

WHC significantly influences the shelf life and sensory acceptance of fermented milk products. The WHC of the prepared quinoa fermented milk was 45.27% on the first day of storage and significantly increased during storage, reaching 52.19% by the 14th day (Figure 11C). The enhancement in WHC during fermented milk storage can be attributed to the synergistic effects of multiple factors. Firstly, casein in fermented milk forms a network structure during fermentation to encapsulate water and enhance water retention. Over time, with the decrease in pH and increase in acidity of the fermented milk, casein is re-aggregated to form a stable gel state, which is conducive to water retention. Secondly, due to the low storage temperature of fermented milk, the aggregation of casein is intensified during refrigeration, which further increases WHC. Additionally, the activity of LAB in fermented milk produces exopolysaccharides, which can also increase the apparent viscosity and stability of fermented milk, thereby enhancing its WHC [57]. This research also assessed the suspension stability of quinoa fermented milk during storage, with a lower R value indicating better suspension stability of the fermented milk. As depicted in Figure 11C, the suspension stability gradually increased with an extension of the storage time, with the optimal effect being observed on the 14th day.

This study also observed changes in the color of quinoa fermented milk during the storage process. The L*, a*, and b* values all increased with the storage time (Figure 11D). A higher L* value indicates a higher product brightness [58]. It has been reported that a decrease in L* is associated with reactions such as lipid oxidation, and a difference of more than 0.5 between a* and b* exceeds the limit of sensory perceptibility [3]. The results of this study show that the difference between the a* and b* values of quinoa fermented milk during storage was much greater than 0.5. In conclusion, the quinoa fermented milk showed some degree of stability, but its characteristics varied over time, especially in terms of acidity.

## 4. Conclusions

In summary, the quinoa fermented with different LAB strains exhibited a decreasing trend in protein, starch, dietary fiber, bound phenolic, and saponin contents, while significantly increasing in the content and antioxidant activity of free phenolics. A comprehensive evaluation by PCA identified *MS2* (a 1:1:1 mixture of *L. casei89*, *L. fermentum61*, and *L. rhamnosus05*) as a high-quality quinoa fermenting agent. Moreover, quinoa fermented milk prepared with *MS2* showed a favorable fruity, caramel, floral, and mildly sweet flavor. After 21 days of storage, the quinoa fermented milk could well maintain the viable bacteria count above 10 log CFU/mL, as well as a high WHC and suspension stability. This research offers a theoretical foundation and empirical data to support the development of quinoa fermented milk characterized by high probiotic activity, distinct flavor profiles, and good storage stability.

## Figures and Tables

**Table 1:** Experiment design of inoculation proportions.

Group	Inoculation Ratio	Quinoa Mass (g)	Volume of Bacterial Suspension (mL)						
			*LB*	*LC*	*LF*	*LR*	*LP*	*LA*	Sterile Water
*CK*	—	100	0	0	0	0	0	0	30
*LB*	*Lactobacillus bulgaricus*	100	5	0	0	0	0	0	25
*LC*	*Lactobacillus casei*	100	0	5	0	0	0	0	25
*LF*	*Lactobacillus fermentum*	100	0	0	5	0	0	0	25
*LR*	*Lactobacillus rhamnosus*	100	0	0	0	5	0	0	25
*LP*	*Lactobacillus plantarum*	100	0	0	0	0	5	0	25
*LA*	*Lactobacillus acidophilus*	100	0	0	0	0	0	5	25
*MS1*	*LA*:*LP*:*LB* 1:1:1	100	1.667	0	0	0	1.667	1.667	25
*MS2*	*LR*:*LF*:*LC* 1:1:1	100	0	1.667	1.667	1.667	0	0	25
*MS3*	*LA*:*LP*:*LB*:*LR*:*LF*:*LC* 1:1:1:1:1:1	100	0.833	0.833	0.833	0.833	0.833	0.833	25
*MS4*	*LR*:*LF*:*LC*:*LP* 1:1:1:1	100	0	1.25	1.25	1.25	1.25	0	25

Note: CK = unfermented quinoa (control group). *LB*, *LC*, *LF*, *LR*, *LP*, *LA*, *MS1*, *MS2*, *MS3*, *MS4* = fermented quinoa (different treatment groups).

**Table 2:** Effects of fermentation with various LAB strains on quinoa color.

Group	L*	a*	b*	ΔE
*CK*	68.63 ± 0.17 ^a^	3.67 ± 0.15 ^f^	12.32 ± 0.62 ^bcd^	
*LB*	58.90 ± 0.05 ^c^	5.24 ± 0.29 ^cd^	12.97 ± 0.28 ^bc^	9.88
*LC*	60.31 ± 0.08 ^b^	4.62 ± 0.05 ^e^	11.99 ± 0.02 ^cd^	8.38
*LF*	53.50 ± 1.15 ^g^	6.13 ± 0.40 ^ab^	11.51 ± 0.23 ^d^	15.35
*LR*	54.39 ± 0.32 ^f^	6.21 ± 0.19 ^ab^	12.46 ± 0.65 ^bcd^	14.47
*LP*	59.85 ± 0.20 ^b^	5.02 ± 0.15 ^d^	12.01 ± 0.53 ^cd^	8.89
*LA*	56.64 ± 0.11 ^d^	5.52 ± 0.24 ^c^	12.46 ± 0.63 ^bcd^	12.13
*MS1*	54.92 ± 0.76 ^f^	6.15 ± 0.16 ^ab^	12.78 ± 0.99 ^bcd^	13.94
*MS2*	54.79 ± 0.29 ^f^	5.60 ± 0.07 ^c^	12.29 ± 0.50 ^bcd^	13.97
*MS3*	55.69 ± 0.17 ^e^	5.95 ± 0.03 ^b^	13.32 ± 0.60 ^ab^	13.18
*MS4*	56.41 ± 0.13 ^de^	6.37 ± 0.22 ^a^	13.97 ± 0.21 ^a^	12.62

Note: *CK*, unfermented quinoa (control group). *LB*, *LC*, *LF*, *LR*, *LP*, *LA*, *MS1*, *MS2*, *MS3*, and *MS4* represent fermented quinoa (different treatment groups). L*, lightness; a*, green and redness; b*, blue and yellowness; ΔE, color variation. Different letters within the same column denote statistically significant differences (*p* < 0.05).

## Data Availability

The data presented in this study are available in the article.

## Supplementary Materials

The following supporting information can be downloaded at: https://www.mdpi.com/article/10.3390/foods14081406/s1, Figure S1: The sensory score of quinoa fermented milk; Table S1: Sensory evaluation criteria; Table S2: Characteristic value and accumulative contribution rate of each principal component; Table S3: Quality load matrix of quinoa fermented by different LAB strains; Table S4: Comprehensive quality evaluation of quinoa fermented by different LAB strains; Table S5: Identification of volatile compounds in quinoa fermented milk using GC-IMS.
