# Supplementary material for: Enhancement of Nutritional Value and Sensory Characteristics of Quinoa Fermented Milk via Fermentation with Specific Lactic Acid Bacteria
Source: Foods. 2025 Apr 18;14(8):1406. doi: 10.3390/foods14081406 (PMC12026847; doi:10.3390/foods14081406)
Supplement: Supplementary file 1 [file foods-14-01406-s001.zip › foods-3577465-supplementary materials.pdf]

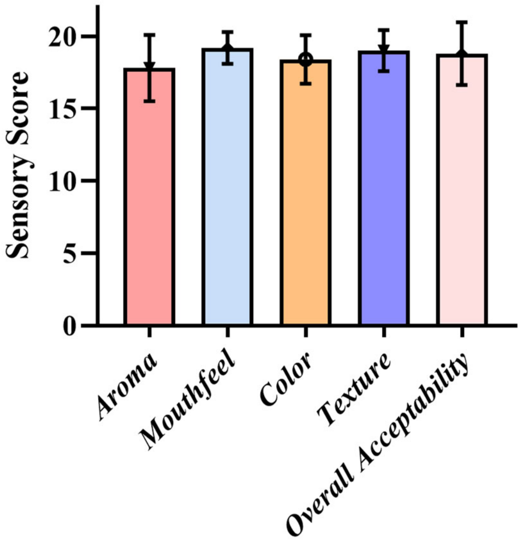

Figure S1. The sensory score of quinoa fermented milk

Table S1 Sensory evaluation criteria

Sensory index	Standard for evaluation	Score
Aroma	Roasted fragrance, natural fermented yogurt smell, harmonious scent	12-20
	Roasted fragrance, quinoa aroma not prominent	6-11
	No quinoa aroma, off-flavors such as sour smell	0-5
Mouthfeel	Smooth and creamy, no pasty mouthfeel, typical yogurt flavor	16-20
	Moderate acidity, acceptable taste	8-15
	Imbalanced sweet and sour, rough texture, strong pasty mouthfeel	0-7
Color	Uniform color, high gloss, light brown color	12-20
	Uniform color, high gloss, brown slightly insufficient	6-11
	Uneven color, inharmonious appearance, brown too light or too dark	0-5
Texture	Uniform texture, no layering, no flocculation, no bubbles, no whey separation	16-20
	Fairly uniform texture, no layering	10-15
	Slight layering, with flocculation, bubbles present	6-9
	Coarse texture, visible particles, obvious layering	0-5
Overall Acceptability	Overall satisfaction, acceptable	10-20
	Overall average, acceptable	5-11
	Overall poor, unacceptable	0-4

Table S2 Characteristic value and accumulative contribution rate of each principal component

Component	Initial Eigenvalues			Extraction Sums of Squared Loadings		
	Total	Variance contribution/%	Accumulation/%	Total	Variance contribution/%	Accumulation/%
1	4.85	34.644	34.644	4.85	34.644	34.644
2	2.824	20.169	54.813	2.824	20.169	54.813
3	1.998	14.268	69.081	1.998	14.268	69.081
4	1.485	10.605	79.685	1.485	10.605	79.685
5	1.266	9.042	88.728	1.266	9.042	88.728
6	0.726	5.185	93.913			
7	0.327	2.338	96.251			
8	0.167	1.193	97.443			
9	0.153	1.094	98.538			
10	0.111	0.795	99.332			
11	0.045	0.319	99.651			
12	0.033	0.237	99.889			
13	0.013	0.095	99.983			
14	0.002	0.017	100			

Table S3 Quality load matrix of quinoa fermented by different LAB strains

Quality indices	Component matrix				
	1	2	3	4	5
Free phenolics	0.569	-0.722	0.175	0.128	-0.155
Bound phenolics	0.689	-0.258	0.373	-0.378	0.003
Saponins	0.594	-0.246	-0.353	-0.107	-0.118
Scavenging DDPH·	0.551	0.332	0.439	-0.439	-0.387
Scavenging ABTS·	0.855	0.311	-0.108	0.247	-0.194
Albumin	-0.796	0.074	0.291	0.447	0.024
Globulin	0.143	-0.321	0.462	-0.117	0.779
Dietary fiber	0.227	0.411	-0.839	0.023	0.036
Amylose	0.842	0.06	-0.201	0.126	0.4
Amylopectin	0.832	0.241	-0.171	0.195	0.343
Total sugar	0.287	-0.283	0.195	0.82	-0.226
Color	-0.257	-0.657	-0.41	-0.325	-0.181
Total phenolics after digestion	0.511	0.58	0.464	0.038	-0.226
Bioaccessbility	-0.383	0.883	0.073	-0.113	0.141

Table S4 Comprehensive quality evaluation of different LAB strains fermented quinoa

Strains	PC score					Comprehensive score (F)	Sort
	1	2	3	4	5		
LB	-2.46	-2.15	-1.357	1.36	-1.12	-1.50	10
LC	-1.58	0.29	-1.22	-2.69	-1.07	-0.79	9
LF	-1.07	2.59	1.88	1.26	-1.58	0.29	4
LR	-2.99	2.35	-0.27	0.76	2.20	-0.40	7
LP	0.23	-1.49	-0.88	0.76	0.77	-0.27	6
LA	-1.03	-0.99	0.38	0.63	0.24	-0.47	8
MS1	2.04	-0.31	-1.04	0.44	0.28	0.52	3
MS2	4.02	1.39	-0.50	0.50	0.41	1.64	1
MS3	2.45	0.44	-0.01	-0.97	-0.80	0.84	2
MS4	0.38	-2.36	3.01	-0.96	0.67	0.14	5

Table S5 Identification of volatile compounds in quinoa yogurt based on GC-IMS

Count	Compound	Formula	MW	RI	Rt [sec]	Dt [a.u.]
1	Durene	$C_{10}H_{14}$	134.2	1482.9	1100.275	1.224
2	Acetic acid	$C_2H_4O_2$	60.1	1443.8	971.023	1.15555
3	Piperazine	$C_4H_{10}N_2$	86.1	1417	891.519	1.08799
4	1	*	0	1420.1	900.202	1.05087
5	2-Nonanone-M	$C_9H_{18}O$	142.2	1366.2	757.976	1.401
6	2-Nonanone-D	$C_9H_{18}O$	142.2	1365.5	756.165	1.87861
7	1 -hexanol	$C_6H_{14}O$	102.2	1349.1	717.671	1.32765
8	2	*	0	1337.6	691.671	1.10442
9	Dipropyl disulfide	$C_6H_{14}S_2$	150.3	1368.7	763.941	1.47482
10	2-Butanone, 3-hydroxy-M	$C_4H_8O_2$	88.1	1299.8	612.989	1.06259
11	2-Butanone, 3-hydroxy-D	$C_4H_8O_2$	88.1	1299.8	612.989	1.33176
12	1 -hydroxy-2-propanone	$C_3H_6O_2$	74.1	1297.8	609.192	1.23576
13	1-octanal	$C_8H_{16}O$	128.2	1299.3	612.04	1.40706
14	3	*	0	1275.6	569.832	1.97613
15	2-Heptanone-M	$C_7H_{14}O$	114.2	1186.3	437.214	1.26424
16	2-Heptanone-D	$C_7H_{14}O$	114.2	1182.1	431.793	1.63264
17	Thiazole	C_3H_3NS	85.1	1257.8	540.558	1.25745
18	4	*	0	1294.2	602.136	1.55294
19	2-Hexenal	$C_6H_{10}O$	98.1	1255	536.069	1.17983
20	(E)-2-hexen-1-al	$C_6H_{10}O$	98.1	1199.4	454.478	1.5088
21	1-hexanal	$C_6H_{12}O$	100.2	1082.9	305.695	1.27185

22	5	*	0	1052.1	276.591	1.80948
23	2,3 Butanedione-M	$C_4H_6O_2$	86.1	988	224.649	1.0383
24	2,3 Butanedione-D	$C_4H_6O_2$	86.1	988.8	225.255	1.17076
25	sec-butyl acetate	$C_6H_{12}O_2$	116.2	988	224.649	1.21491
26	2-Pentanone	$C_5H_{10}O$	86.1	988.5	224.984	1.37153
27	Pyrrolidine	C_4H_9N	71.1	1013.9	244.316	1.26808
28	Ethanol	C_2H_6O	46.1	938	192.252	1.1251
29	2-Butanone	C_4H_8O	72.1	917.7	180.495	1.24531
30	Butanal-D	C_4H_8O	72.1	911.1	176.856	1.2863
31	Acetic acid ethyl ester	$C_4H_8O_2$	88.1	901	171.39	1.33694
32	2-methyl-2-propenal	C_4H_6O	70.1	896.1	168.795	1.22541
33	Acetone	C_3H_6O	58.1	851.7	147.003	1.11671
34	triethylamine	$C_6H_{15}N$	101.2	800.1	125.211	1.09412
35	6	*	0	855.1	148.56	1.04894
36	Butanal-M	C_4H_8O	72.1	902	171.909	1.11388
37	cyclohexane	C_6H_{12}	84.2	772.3	114.834	1.13365
38	7	*	0	849.5	145.998	1.60583
39	8	*	0	882.5	161.794	1.47228
40	9	*	0	811.9	129.891	1.39811
41	n-Pentanal	$C_5H_{10}O$	86.1	980.4	219.373	1.42746
42	2-Hexanone	$C_6H_{12}O$	100.2	1081	303.814	1.19195
43	2- butanol	$C_4H_{10}O$	74.1	997	231.291	1.32646
44	10	*	0	1010.2	241.431	1.45603

45	Isovaleric acid, methyl ester	$C_6H_{12}O_2$	116.2	1012.2	242.993	1.53364
46	ethyl (E)-2-butenoate	$C_6H_{10}O_2$	114.1	1141.5	374.783	1.18457
47	2-Methylbutyl acetate	$C_7H_{14}O_2$	130.2	1129	358.71	1.2915
48	11	*	0	982.3	220.703	1.25161
